# Silencing of *CYP6* and *APN* Genes Affects the Growth and Development of Rice Yellow Stem Borer, *Scirpophaga incertulas*

**DOI:** 10.3389/fphys.2016.00020

**Published:** 2016-02-12

**Authors:** Vijaya Sudhakara Rao Kola, P. Renuka, Ayyagari Phani Padmakumari, Satendra K. Mangrauthia, Sena M. Balachandran, V. Ravindra Babu, Maganti S. Madhav

**Affiliations:** ^1^Department of Biotechnology, Indian Council of Agricultural Research-Indian Institute of Rice ResearchHyderabad, India; ^2^Department of Entomology, Indian Council of Agricultural Research-Indian Institute of Rice ResearchHyderabad, India; ^3^Department of Plant Breeding, Indian Council of Agricultural Research-Indian Institute of Rice ResearchHyderabad, India

**Keywords:** yellow stem borer-YSB, double stranded RNA (dsRNA), growth and development, bioassays, cytochrome P450 (*CYP6*), aminopeptidase N (*APN*)

## Abstract

RNAi is a powerful tool to target the insect genes involved in host-pest interactions. Key insect genes are the choice for silencing to achieve pest derived resistance where resistance genes are not available in gene pool of host plant. In this study, an attempt was made to determine the effect of dsRNA designed from two genes *Cytochrome P450 derivative* (*CYP6*) and *Aminopeptidase N* (*APN*) of rice yellow stem borer (YSB) on growth and development of insect. The bioassays involved injection of chemically synthesized 5′ FAM labeled 21-nt dsRNA into rice cut stems and allowing the larvae to feed on these stems which resulted in increased mortality and observed growth and development changes in larval length and weight compared with its untreated control at 12–15 days after treatment. These results were further supported by observing the reduction in transcripts expression of these genes in treated larvae. Fluorescence detection in treated larvae also proved that dsRNA was readily taken by larvae when fed on dsRNA treated stems. These results from the present study clearly show that YSB larvae fed on dsRNA designed from *Cytochrome P450* and *Aminopeptidase N* has detrimental effect on larval growth and development. These genes can be deployed to develop YSB resistance in rice using RNAi approach.

## Introduction

The rice yellow stem borer (YSB), *Scirpophaga incertulas* (Lepidoptera: Crambidae), is a widely distributed and destructive insect that causes significant yield losses to an extent of 10–90% by feeding on rice crop at all stages (Bandong and Litsinger, 2005). The YSB larvae that enter inside the stem cause death of the central shoot known as “dead hearts” at vegetative stage and unfilled panicles known as “white heads” at reproductive stage (Pathak and Khan, 1994). There are no resistant sources available for this deadly pest in cultivated rice germplasm (Brar and Khush, 1997). Although the option of expression of cry genes from *Bacillus thuringiensis* (Bt) is available, development of insect resistance in field has become one of the long standing issue with Bt food crops (Tabashnik et al., 2013). Given these circumstances, silencing of YSB genes through RNA interference (RNAi) by inactivating the key insect genes leading to aberration in pest growth and metabolism (Yang et al., 2011) offers an alternative. RNAi is sequence-specific gene silencing at the post-transcription level, induced by double stranded RNA (dsRNA; Fire et al., 1998; Hamilton et al., 2002). Down regulation of the expression of specific target genes through RNAi has been widely used for genetic research in several insects (Price and Gatehouse, 2008). However, selection of crucial genes which have an important role in insect life cycle for a specific insect is still a challenge (Kola et al., 2015).

Cytochrome P450 monooxygenases (cytochrome P450s) are found in virtually all living organisms. It plays an important role in the metabolism of xenobiotics such as drugs, pesticides, and plant toxins (Scott, 2008). In insects, cytochrome P450s play a predominant role in the metabolism of insecticides, which often results in the development of insecticide resistance in insect populations (Zhou et al., 2010). Most insect cytochrome P450 genes belong to microsomal *CYP4, CYP6, CYP9, CYP28, CYP321*, and mitochondrial *CYP12* families which have been associated with detoxification processes allowing the insect to become tolerant or resistant to insecticides or host plant allelochemicals (Guo et al., 2011). Aminopeptidase N (*APN*) is a member of metallo enzymes present in larvae midgut of lepidopteran insects. It plays an important physiological role in dietary protein digestion (Marchler-Bauer et al., 2015). Inhibition of its activity in the midgut can result in detrimental effect on larval growth, development, and lead to larval mortality (Reed et al., 1999). *APNs* are mainly studied for their role as receptors in Cry toxin-induced pathogenesis in insects (Bravo et al., 2007). Expression of *APN*s was found in midgut and malphigian tubules (Wang et al., 2005). RNAi mediated silencing of *CYP6AE14* (Cytochrome P450 derivative) and *APN* in other lepidopteran insects like *Helicoverpa armigera, Plutella xylostella, Achaea janata*, and *Spodoptera litura* resulted in significant down-regulation of corresponding transcript and protein expression causing larval growth arrest and mortality and development of lethal larval-pupal intermediates (Rajagopal et al., 2002; Mao et al., 2007; Sivakumar et al., 2007; Bautista et al., 2009; Ningshen et al., 2013).

The present work was aimed to examine the effect of dsRNA molecules on silencing of YSB genes *CYP6* and *APN* by feeding larvae on dsRNA treated cut stems. The dsRNAs fed larvae were examined to see the effect of silencing of these genes on insect growth and metabolism. Our bioassays simulated the *in vivo* mechanism of gene silencing and showed that dsRNA molecules can be taken up through the normal dietary path of YSB. Further, the target gene expression level was examined in control and dsRNA fed larvae which suggested silencing of the corresponding gene of YSB which is the first RNAi approach for YSB.

## Materials and methods

### Design of double stranded RNA and prediction of off targets

**Figure 1:**
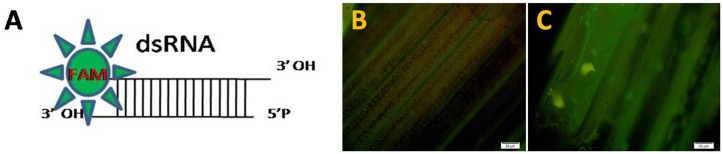
**(A)** Schematic representation of dsRNA labeled with 5 FAM, Fluorescence detection of dsRNA in TN1 cut stems under microscope **(B)** Untreated **(C)** Treated cut stem.

To clone cDNAs of *CYP6* and *APN* genes, a total of 48 corresponding sequences of *CYP6* gene were downloaded from lepidopteron database, NCBI. Out of these, 10 sequences could find near homologies which were used for designing three primers. Similarly, to clone *APN*, total 47 sequences were downloaded from lepidopteron database, NCBI. Out of these, 25 sequences showed near homologies which were used to design two sets of primers from the conserved region through online tool (http://primer3.ut.ee/). Using these primers 750 bp cDNA of *CYP6* and 728 bp cDNA for *APN* were cloned from YSB and submitted to *Genbank* (*CYP6*: KC904274, *APN*: KF290773). These YSB cDNA sequences were used for identification of siRNA hotspots and dsRNAs were designed based on the Reynold rules (Reynolds et al., 2004). The designed dsRNA for *CYP6* sense strand was 5′-AGUUGAGAAUGAAAUGACUGA-3′ and the antisense was 3′-UCAACUCUUACUUUACUGACU-5′ which had high Reynolds score of six out of eight. The similar strategy was followed for designing dsRNA from *APN* gene, the designed dsRNA sense strand sequence was 5′ GACGACGUAUACUUAACUACU-3′ and the antisense was 3′-CUGCUGCAUAUGAAUUGAUGA-5′ which had the high Reynolds score eight out of eight. Care was taken for designed dsRNA not to have any unintended off targets, which was apparent by doing BLAST search. The designed dsRNAs were also tested in *in silico* to ascertain the fulfillment of different parameters for maximum silencing by using siMAX siRNA design tool (https://www.eurofinsgenomics.eu/en/dna-rna-oligonucleotides/custom-rna-oligos/simax-sirna.aspx). The selected dsRNA were chemically synthesized at Eurofins Genomics GmbH, Germany by attaching FAM (5′carboxy fluorescein) at 5′ position (Figure 1A).

### Bio assay

Adult moths of YSB were collected from the rice field at Indian Institute of Rice Research (IIRR), and released on susceptible *cv*. TN1 plants in glasshouse for egg laying. These egg masses were collected and transferred into plastic vials for hatching. The neonate larvae that hatched from a single egg mass were released on to ~8 cm long TN1 stems and reared at 25 ± 2°C for 3 days (Padmakumari et al., 2013). For *in vitro* cut stem bioassay, fresh TN1cut stems (~8 cm) were placed in petri plate with moistened filter paper. For standardization of optimum concentration of dsRNA required for assay, three different concentrations i.e., 10, 20, and 30 pM of dsRNA were chosen. The dsRNA was injected into cut stems using small insulin syringe with volume of 30 μl per stem. Buffer (1 ×) of dsRNAs was used as control. The cut stems were kept at room temperature for 1 h for dsRNA to spread completely within the stem. On each treated cut stem, three larvae of 3 day old were released with the help of a soft paint brush. The insects were reared at 25 ± 2°C, 65%RH and 16:8 h (L: D). The optimized 30 pM concentration of dsRNA was used to determine its effect on larva, initially the experiment was carried out with different dsRNA concentration *viz*., 10, 20, 30, and 40 pM. (Figures showing the effect of these concentrations on YSB were submitted as Supplementary Material). We found that the response was good at 30 pM. Hence in all the experiments we used 30 μl of 30 pM dsRNA per 8 cm cut stem to evaluate the effect on the target insect. Each bioassay was carried with 15 stems in three replication (*N* = 45) and each experiment repeated twice to obtain uniform results. Various parameters like insect molting, abnormal morphological changes in the larval growth and length, and survival were observed at 6, 12, 15 days after treatment (DAT). At each time interval, three samples (nine larvae) were selected for observation by opening stems through destructive sampling. The dead larvae were dissected to observe under fluorescent microscope (Olympus CH40, Japan).

### Effects of dsRNA on YSB larval growth and development

#### Larval mortality

The mortality of larvae was recorded by opening the cut stems under a microscope at 3 day intervals before changing on to fresh treated stems. The number of surviving larvae per concentration of dsRNA was recorded at different time intervals of 6, 12, and 15 days after treatment. The mortality of the larvae in both treated and untreated stems were recorded.

#### Larval weight and length

Reduction in larval weight was obtained by calculating differences in the mean of larval weight in dsRNA treated samples with that of untreated control. Larval weight was measured in milligrams using an electronic balance and larval size with centimeter scale.

#### Fluorescent detection of dsRNA

To confirm the dsRNA uptake by larvae which were fed on treated stems, fluorescence detection was conducted using a fluorescent microscope (Olympus CH40, Japan) in the range of 460–480 nm. To detect the dsRNA in whole larva as well as in the larval midgut, florescence was observed at 6, 12, and 15 days after treatment.

#### Statistical analysis

Data on larval length, larval weight and percentage of larval mortality for *CYP6* and *APN* were analyzed by factorial ANOVA using Statistix 8.1 (Analytical Software, 2005). We observed that variance of mortality among the time intervals was slightly unequal, to remove this effect, percent mortality was arc sine transformed, this transformation was made following rules mentioned in Gomez and Gomez (1984).

#### Determination of silencing

To detect the silencing of *CYP6* and *APN* in treated larvae, total RNA was extracted using Trizol reagent from 4 mg of single larvae. cDNA was synthesized according to manufacturer's instructions by using Prime Script™ cDNA Synthesis Kit (Takara, Japan) from the total RNA extracted at various time intervals. The Quantitative Real time PCR (qRT-PCR) reactions were carried out with SYBR Premix Ex TaqTM (Takara Bio INC, Japan) following the manufacturer's protocol using a Real time PCR LC-96 (Roche LightCycler® 96). The qRT-PCR primers were designed using the online tool Primer3 (http://primer3.ut.ee/). The primers used were *CYP6* Forward-GATTTT CGACGTTACCCTCG Reverse- CCGCTGGGTTGGTAATTCC and *APN* Forward- AGGATTCAAGAGCTGGTCGT Reverse- GATGACTTCGGTGTGAGGCA. For internal control, endogenous genes 18s rRNA from the Lepidopteron database and β-Actin were used (Kumar et al., 2009). Standard qRT-PCR procedures were followed with annealing of 60°C for 30 s. The specificity of the PCR reactions was monitored with melting curve analysis and gel electrophoresis. The relative gene expression data were analyzed by LC96 qPCR and by using the 2^−ΔΔCt^ method as described previously (Schmittgen and Livak, 2008).

### Effect of dsRNA on pink stem borer (PSB)

To check the specificity of RNAi effect and off-target effect of dsRNA, the designed dsRNAs were tested on rice pink stem borer (*Sesamia inferens*) through *in vitro* bioassay. Bioassays were performed as like YSB and observation were taken on larval length and weight. The data was analyzed statistically by *T*-test using Statistix 8.1 software (Analytical Software, 2005).

## Results

### Cloning of cDNAs from YSB and designing dsRNA

In this study, systematic efforts were made to demonstrate the silencing of *CYP6* and *APN* genes in YSB through *in vitro* studies using rice cut stems injected with corresponding dsRNAs. cDNAs of *CYP6* and *APN* were cloned, *CYP6* cDNA of YSB showed 70% identity with *Chilo suppressalis*, 69% with *Spodoptera*, 64% with *Helicoverpa*, and only 69% with Rice Leaf roller Cnaphalocrocis medinalis. Similarly, *APN* of YSB showed 71% identity with *Chilo suppressalis*, 89% with *Spodoptera*, 82 % with *Helicoverpa*, and very less 69% with Rice Leaf roller Cnaphalocrocis medinalis. Among the siRNA hotspot regions, the region which does not have any homology to the related species was selected to design 21-nucleotide dsRNA. The designed two dsRNA sequences did not match to the any of the related species as well with mammalian species available in Genbank which indicates that it does not have the off targets. The cut stem assay was designed so as to simulate a real time situation wherein the larva bore the stem pieces and enter into stem. As YSB is a monophagous pest of paddy, we used cut stems of TN1 (highly susceptible variety) for standardization of dsRNA concentration. We found 30 pM concentration of dsRNA could effectively silence the *CYP6* and *APN* genes and showed reduced growth and development, whereas, 10 and 20 pM concentration of dsRNA showed very less effect on larval growth (Supplementary Figure 1).

### Effect of dsRNA on growth and development

**Figure 2:**
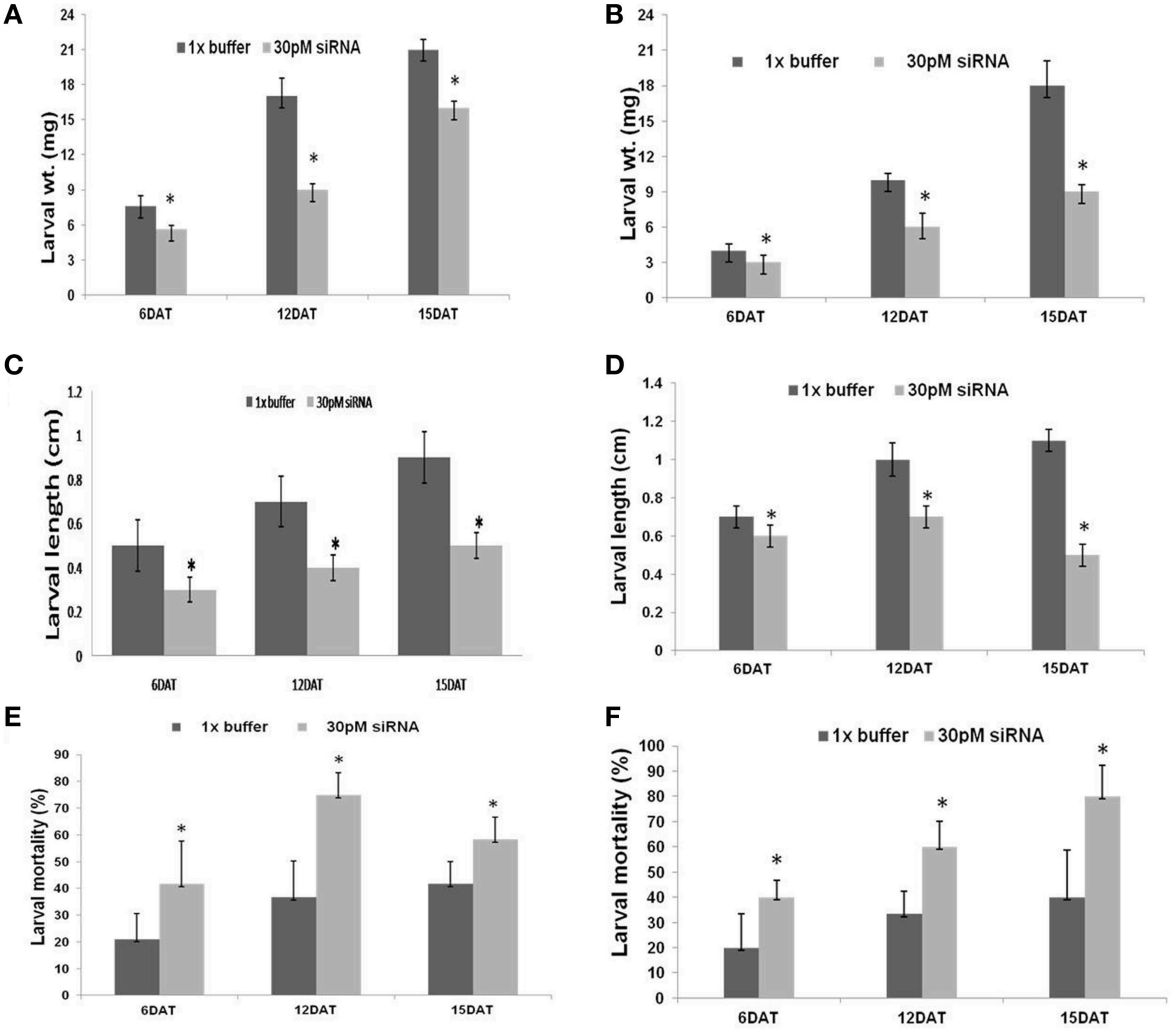
**(A)** Effect of *CYP6* 30 pM dsRNA on YSB larval weight at different time intervals, ^*^Indicates that larval weight in treatments were significantly different (*N* = 3, *F* = 60.27, DF = 11, *P* < 0.001) from larval weight in control. DAT -days after treatment. **(B)** Effect of *APN* 30 pM dsRNA on YSB larval weight at different time intervals, ^*^Indicates that larval weight in treatments were significantly different (*N* = 3, *F* = 28, DF = 12, *P* < 0.002) from control, DAT- days after treatment. **(C)** Effect of *CYP6* 30 pM dsRNA on YSB larval length at different time intervals, ^*^Indicates that larval lengths in treatments were significantly different from control larval length. **(D)** Effect of *APN* 30 pM dsRNA on YSB larval length at different time intervals, ^*^Indicates that larval length were significantly different (*N* = 3, *F* = 35.64, DF = 14, *P* < 0.0001) from larval length in control. **(E)** Effect of *CYP6* 30 pM dsRNA on YSB larval mortality at different time intervals, ^*^Indicates that larval mortality in treatments were significantly different (*N* = 4, *F* = 9.13, DF = 18, *P* < 0.007) from control larval mortality. **(F)** Effect of *Amino* 30 pM dsRNA on YSB larval mortality at different time intervals,^*^Indicates that larval mortality were significantly different (*N* = 5, *F* = 12.73, DF = 24, *P* < 0.0016) from control larval mortality.

The dsRNA of *CYP6* and *APN* had a pronounced effect on growth and development of the larvae. Larvae fed with dsRNA specific to the *CYP6* and *APN* showed significant inhibition of growth in a time dependent manner. Treated larvae (dsRNA) showed significant reduction in growth and development characters like larval length and weight compared with untreated larvae at regular time intervals. Larvae fed on *CYP6* dsRNA showed maximum weight reduction (8 mg) at 12 DAT and minimum was at 6 DAT as compared to its untreated control (Figure 2A). dsRNA designed from *APN* also showed significant larval weight and length reduction compared to its untreated larvae. The larval weight reduction was maximum at 15 DAT (Figure 2B). There was a significant reduction in larval length with increase in exposure to dsRNAs. Treated larvae showed a mean reduction in length of 0.45 cm at 15 DAT, 0.3 cm at 12 DAT, and 0.25 cm at 6 DAT compared to untreated control larvae (Figures 2C,D).

### Effect of dsRNA on larval mortality

**Figure 3:**
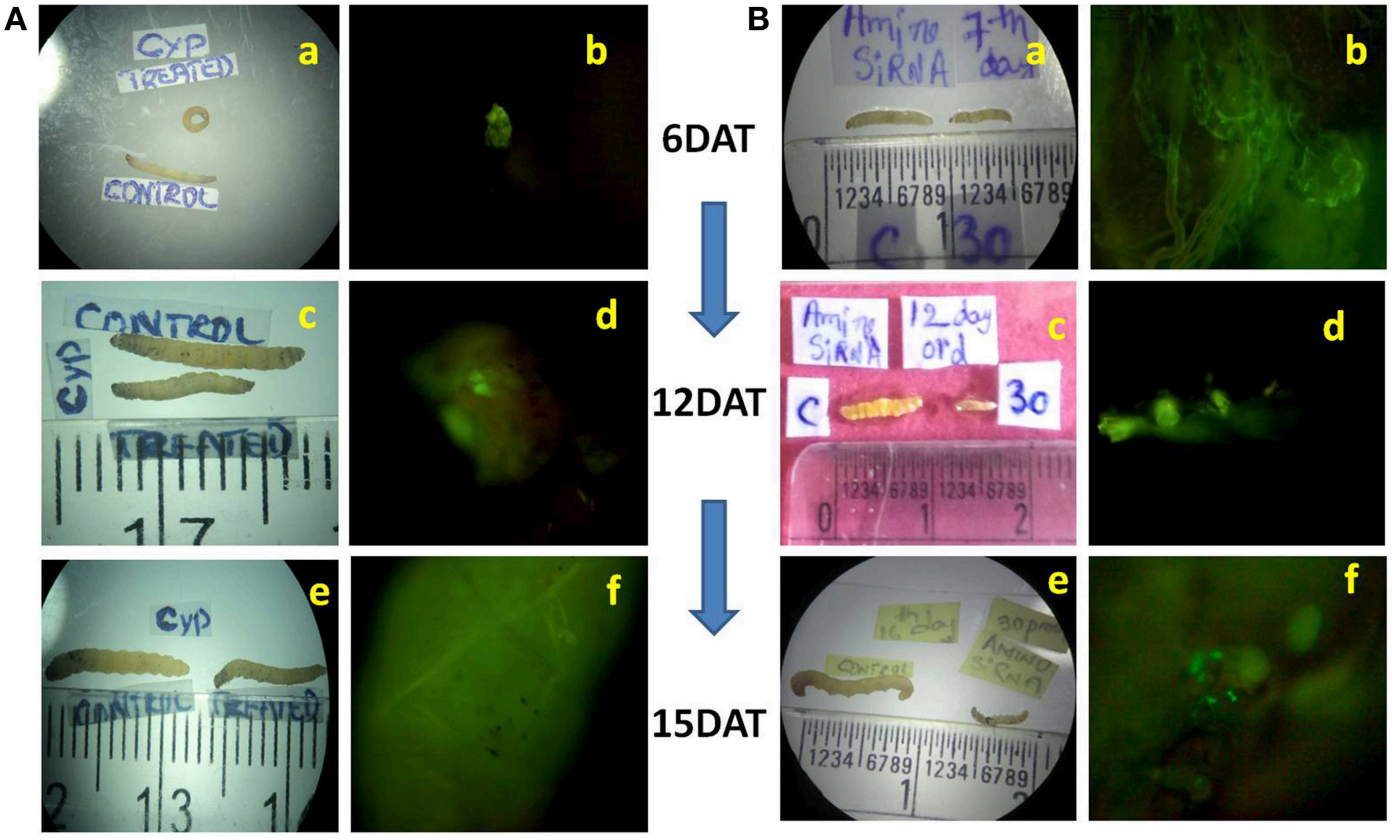
**(A)** Effect of *CYP6* dsRNA on YSB larvae fed on treated TN1 cut stems, Growth reduction in treated larvae at **(a)** 6th DAT, **(c)** 12th DAT **(e)** 15th DAT after feeding on dsRNA treated TN1 cut stems. Presence of fluorescence in larval midgut indicating that dsRNA was ingested by larvae when fed on treated stems at **(b)** 6th DAT, **(d)** 12th DAT, **(f)** 15th DAT. **(B)** Effect of *Amino* dsRNA on YSB larvae fed on treated TN1 cut stems. Growth reduction in treated larvae at **(a)** 6th DAT, **(c)** 12th DAT **(e)** 15th DAT after feeding on dsRNA treated TN1 cut stems. Presence of fluorescence in larval midgut at **(b)** 6th DAT, **(d)** 12th DAT, **(f)** 15th DAT.

Mortality of treated larvae in both dsRNAs increased from 6 to 15 DAT. The highest mortality rate of 74.95% was observed at 12 DAT in *CYP6* (Figure 2E) and 80% at 15 DAT in *APN* treatment (Figure 2F; Supplementary Tables 1A,B). Florescent microscope observation before and after the release of larvae in the treated and untreated stems confirms that the dsRNA abundance and translocation within the injected stem (Figures 1B,C; Supplementary Figure 2). *CYP6* dsRNA fed larvae showed the presence of fluorescence in larval midgut at 6, 12, 15 DAT and it increased with the time course which was also proportional to the increase in larval mortality as well as decrease in larval weight (Figure 3A). Similarly, we found that fluorescence in larval midgut in *APN* dsRNA fed larvae was proportional to the effect on larval growth (Figure 3B). These observations indicate that the designed dsRNA were systemic in nature, and through oral feeding could reach the targets genes, bind to them, thus affecting the larval growth and development.

### Confirmation of gene silencing by qRT-PCR

**Figure 4:**
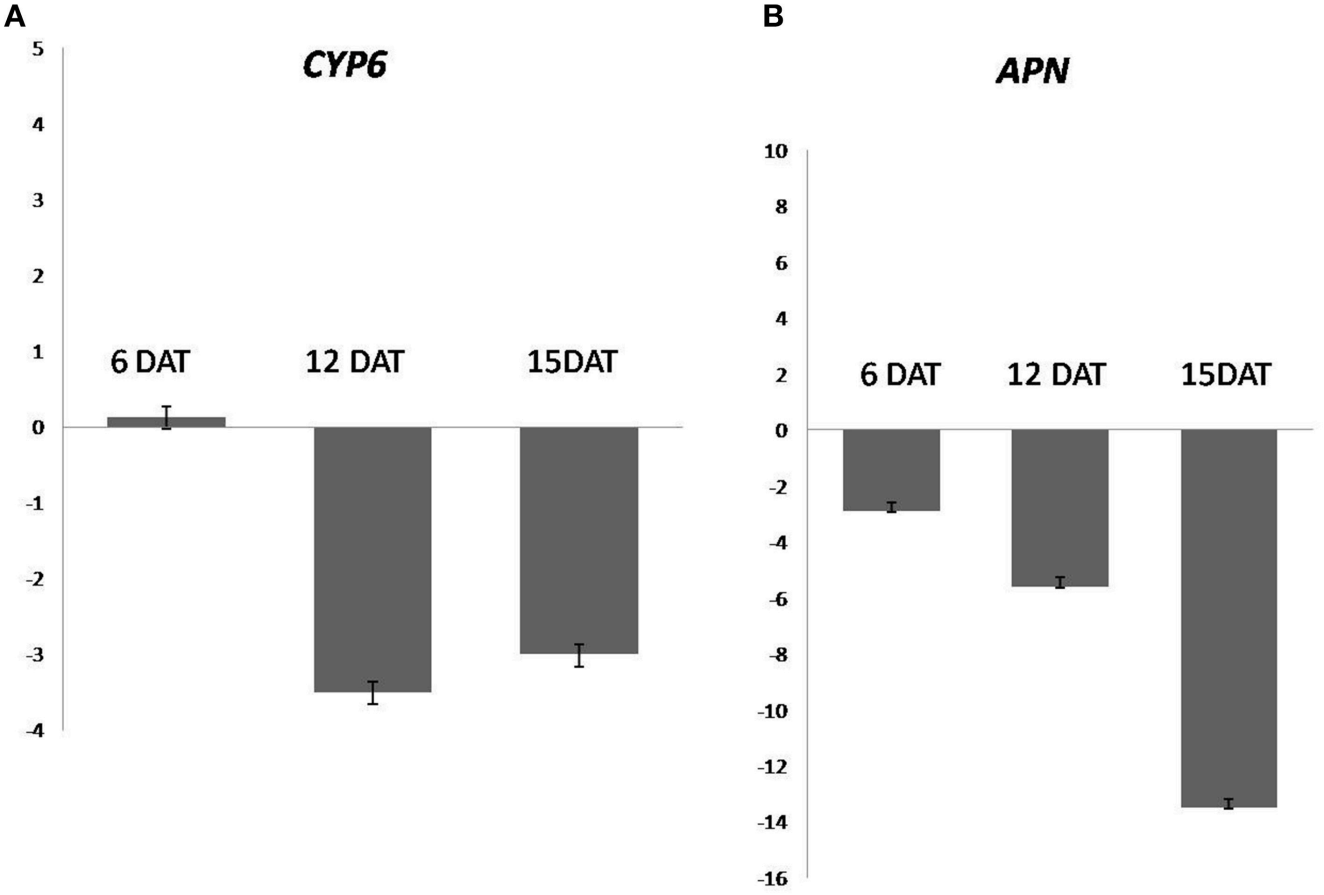
**Relative expression fed on TN1 cut stems treated with *CYP6*-dsRNA and *APN*-dsRNA in an individual bioassays**. β-actin and 18 s were used as an internal controls **(A)** expression of *CYP6* gene from larvae sampled at 6, 12, 15 days after treatment along with respective controls. **(B)** Expression of *APN* in 6, 12, 15 days after treatment along with respective controls.

We have observed the reduction in the expression level of *CYP6, APN* in all the stages of dsRNA treated samples, whereas, maximum silencing was observed at 12 DAT in *CYP6*, and 15 DAT in case of *APN*. Quantitative RT–PCR revealed, *CYP6* expression in treated larvae was reduced by 3.5, 3.0 fold in 12, 15 DAT, respectively. In case of *APN*, the fold change in gene expression was increased by reducing its expression 2.9, 5.6, 13.5 fold in 6, 12, and 15 DAT compared to the control larvae (Figures 4A,B). The reduction in transcript levels in treated samples correlated with bioassay data for *CYP6* and *APN* genes. So these results demonstrated that both genes have key roles in maintaining YSB growth and development.

### Effect of dsRNA on PSB

**Figure 5:**
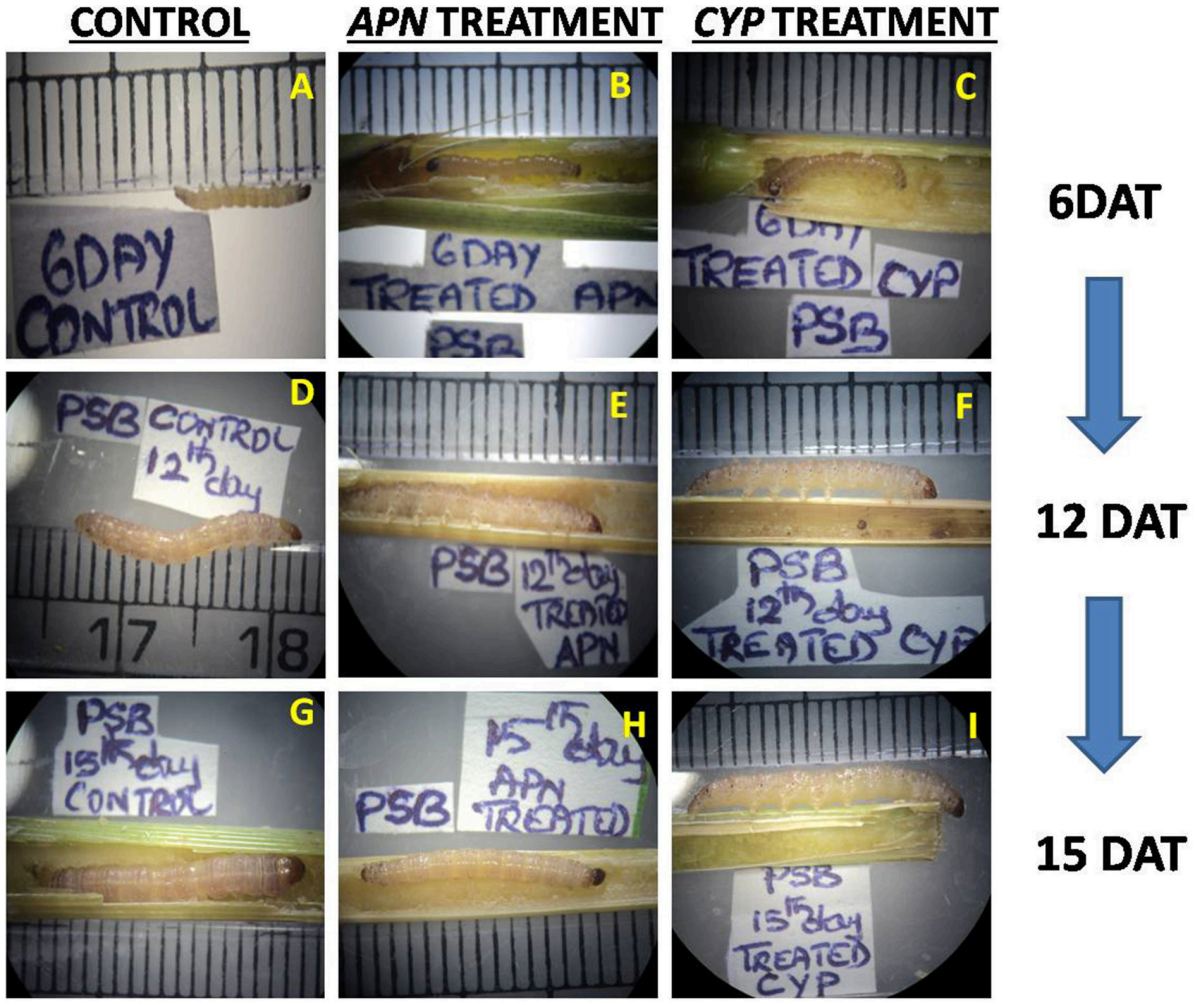
PSB larvae fed on rice TN1 cut stems, 6th DAT **(A)** Control, **(B)** *APN* treated, **(C)** *CYP* treated. 12th DAT **(D)** Control, **(E)** *APN* treated, **(F)** *CYP* treated. 15th DAT **(G)** Control, **(H)** *APN* treated, **(I)** *CYP* treated.

The PSB larvae were reared on the rice TN1 cut stems where YSB specific dsRNAs was injected into the stems. Interestingly, no abnormalities were observed in PSB larvae when fed on treated stems of both dsRNA. In *APN* dsRNA treatment, the PSB larvae lengths were similar to that of untreated control at 6, 12, and 15 DAT. Larval weight (mg) was also similar to the control at 6 and 12 DAT. However, decrease in larval weight was observed at 15 DAT (30 ± 1.0 mg), which is significantly lower (*P*-value 0.0055) compared to untreated control (49.0 ± 1.0 mg) but larval mortality was not observed. Whereas, in case of *CYP6* dsRNA, we did not observe any changes in larval length and weight between control and treatments at three different time intervals. (Supplementary Table 2). Interestingly, larvae were quite active in both the treatments and mortality was not at all observed (Supplementary Data Videos 1–3) and pupae were formed. This clearly indicates that, the designed dsRNAs are specific to the YSB and they did not show any abnormal effect on PSB (Figure 5).

## Discussion

The success of RNAi for insect control depends on identification of suitable genes for RNAi. The candidate gene should not only have insecticidal effects on the target pests, but also safe toward other organisms, natural enemies and human beings (Agarwal et al., 2012; Kola et al., 2015). The present study showed that RNAi technology is an effective strategy for silencing the key genes involved in the metabolic activities of YSB. RNAi-triggered gene suppression through uptake of dsRNAs has been reported for many insect species belonging to Coleoptera, Diptera, Hemiptera, Hymenoptera, Isoptera, Lepidoptera, and Orthoptera (Huvenne and Smagghe, 2010).

In this study, two important genes *viz., CYP6* and *APN* were selected based on their earlier successful reports of silencing in lepidopteron pests (Rajagopal et al., 2002; Mao et al., 2007; Sivakumar et al., 2007; Bautista et al., 2009; Crava et al., 2010; Yang et al., 2010; Zhang et al., 2011; Edi et al., 2014). The Cytochrome P450 monooxygenases are present in almost all organisms including insects. They play vital roles in hormone regulation, metabolism of xenobiotics and in biosynthesis or inactivation of endogenous compounds (Kola et al., 2015; Yu et al., 2015). Similarly, Aminopeptidase N (*APN*s) is primarily involved in dietary protein digestion majorly located at the midgut epithelium. *APNs* are known to cleave a single amino acid residue from the N-terminus of oligopeptides, preferentially the neutral amino acids (Wang et al., 2005; Pigott and Ellar, 2007). As, these two genes are known to play very important role in insect growth and development (Mao et al., 2007; Ningshen et al., 2013), we selected them as a candidate for silencing in YSB. Wang et al. (2015) succeeded in silencing *CYP6AB14* by injecting dsRNA derived from *CYP6AB14* into *S. litura* and found reduced transcript levels of *CYP6AB14* and increased developmental abnormalities and higher mortality rates. Zhang et al. (2013), also observed larval mortality and reduced growth in *H. armigera* upon larval feeding on dsRNA designed from *CYP6B6*. Similar results were also reported with transgenic expression of *CYP6AE14* in tobacco and cotton (Hodgson et al., 1995; Mao et al., 2007, 2011). Recently, Jin et al. (2015) designed dsRNAs targeted to *Chitin synthase* (*Chi*), *CYP6AE14*, and *V-ATPase* genes and expressed in the tobacco chloroplasts for control of *Helicoverpa* and observed reduced transcription of target genes in the insect midgut and stunted larval growth. Silencing of *APN1* was reported in Castor Semilooper, *A. janata* (Ningshen et al., 2013).

For successful design of dsRNAs, there is a need to identify the specific regions or motif of key pest genes. To attain this, for the first time partial length of two candidate genes, *CYP6* and *APN* were cloned from the YSB through PCR based strategy. Though YSB belongs to lepidopteron family, *CYP6* showed 69% identity and *APN* 82% identities with *Helicoverpa* indicating significant differences. The unique regions of two YSB genes were selected for designing dsRNA, following Reynolds rules (Reynolds et al., 2004). The higher scores with these principles suggest having more stability and greater silence effects (Horn et al., 2010). Much of the commercial software currently available for designing dsRNAs follow Reynolds rule, Ui-Tei rule, Amarzguioui rule, and Tuschl rule (Naito and Ui-Tei, 2012). The dsRNAs designed in this study of *CYP6* and *APN* genes had the highest score based on Reynolds rule, which might have positive influence of their higher expression. There were some reports of using long dsRNA for effective RNAi than small dsRNA for insects (Miller et al., 2012; Li et al., 2015), however, recent reports indicated the more effectiveness of small dsRNAs insect control (Kumar et al., 2009; Naito and Ui-Tei, 2012; Gong et al., 2013). Our designed dsRNA from *CYP6* lies in the principle domain of CYP450 group of enzymes, which belongs to haem-thiolate proteins involved in the oxidative degradation of various compounds. This class of proteins has domains viz., haem-binding loop (with an absolutely conserved cysteine that serves as the 5th ligand for the haem iron), the proton-transfer groove and the absolutely conserved EXXR motif in helix K forms a principle domain. Similarly *APN* (zinc-dependent metallopeptidases) is a Type II integral membrane protease belongs to Gluzincin family (thermolysin-like peptidases or TLPs), this family consists of several zinc-dependent metallopeptidases including M1 peptidases and know to consist of a small N-terminal cytoplasmic domain, a single transmembrane domain and a large extracellular ectodomain that contains the active site. The dsRNA designed is lies in the part of active site of this enzyme so we expect that it binds to the target effectively.

Generally insect bioassays have been carried out through introducing dsRNA into an organism by microinjection (Ghanima et al., 2007), soaking and oral feeding through artificial diet (Mao et al., 2007; Chen et al., 2008). Since, YSB is a monophagous pest and does not have artificial diet; we resorted to rearing on rice cut stems, so as to simulate a real time situation where larva bore the stem pieces and enter into stem. In earlier studies cut stem assay were used to assess the toxicity of various toxins and transgenics against YSB (Nayak et al., 1997; Nguyen Huu Ho et al., 2001; Ramesh et al., 2004; Padmakumari et al., 2013). We found lower concentration of dsRNA (30 pM) was effective for silencing of target genes. Kumar et al. (2009) also found that 50 nM concentrations were effective for silencing Acetyl cholinesterase *(Ache)* gene in *H. armigera*. Similarly, Bautista et al. (2009) reported that 250 ng dsRNA targeting *CYP6BG1* delivery through droplet feeding to 4th instar larvae of *Helicoverpa* greatly reduced the *CYP6BG1* expression. To track accurately the presence of dsRNA in various insect tissues, FAM was used to label the dsRNA and confirmed their systemicity in insect tissue. Earlier reports have indicated the use of fluorescent dyes like Fluoroscein isothiocyanate (FITC), Cyanine Cy-3, Cy-5, and FAM to track the movement and binding of dsRNA in the tissues (Urwin et al., 2002; Karim et al., 2010; Hui et al., 2011; Wuriyanghan et al., 2011; Bolognesi et al., 2012; Li et al., 2015).

Our results indicated the increase of YSB larval mortality rates in feeding assay with dsRNA of *CYP6* and *APN* genes. Change in growth and developmental characters were observed from very early larval developmental stages by showing reduced larval growth (larval weight and length), delayed molting and led to death. Interestingly, none of the larvae entered into pupae form. We observed an increase in fluorescence signal with increase in time of exposure which indicates the signal amplification. These observations were correlated with qRT-PCR experiments data.

The designed dsRNA sequences did not match with mammals or related species which are present in GenBank and beneficial insects which suggests that the designed dsRNA molecules may be specific to target insect and may not have unintended effects. Further to make sure of specificity of these molecules, bioassays were carried out in PSB, another stem borer of rice. The results indicated that dsRNAs designed from *CYP6* and *APN* did not showed significant effect on larval length and weight except in case of 15 DAT with *APN* treatment. Further investigations are required to fully understand the delayed response of *APN* treatment in PSB larval weight. There was no morality in both the cases, larvae were quite active completed their instars and entered in to the pupal stage. These results suggest that the YSB dsRNAs of *CYP6* and *APN* genes could not find targets in PSB which further confirms that the designed dsRNAs were more specific to YSB.

In our study, we conclusively proved the key functions of Cytochrome P450 and Aminopeptidase N enzymes in various metabolic pathways of YSB such as normal growth and also its involvement in many stages of development. The present results strongly suggest that these *CYP6* and *APN* genes can be potential targets for insect-control, and insect-resistant transgenic plants may be obtained through RNAi-mediated silencing of insect genes. The off targeting study through *in silico* and *in vivo* showed that the designed dsRNA from these genes are highly specific to insects and do not have any unintended effects on other organisms.

## Author contributions

Conceived and designed the experiment: MSM, SKM, KVSR, PR, APP, SMB, and VRB.

### Conflict of interest statement

The authors declare that the research was conducted by the support of DBT, Govt of India.
