# Supplementary material for: Silencing of CYP6 and APN Genes Affects the Growth and Development of Rice Yellow Stem Borer, Scirpophaga incertulas
Source: Front Physiol. 2016 Feb 12;7:20. doi: 10.3389/fphys.2016.00020 (PMC4751738; doi:10.3389/fphys.2016.00020)
Supplement: Supplementary file 1 [file Table1.DOCX]

**Supplementary table: 1**

**Statistical data Analysis**

**CYP mortality table-A**

| Source | DF | SS | MS | F | P |
| --- | --- | --- | --- | --- | --- |
| V002 | 1 | 2386.32 | 2386.32 | 9.13 | 0.0073* |
| V001 | 2 | 1223.68 | 611.84 | 2.34 | 0.1249 |
| V002* V001 | 2 | 249.87 | 124.93 | 0.48 | 0.6278 |
| Error | 18 | 4706.73 | 261.49 |  |  |
| Total | 23 | 8566.60 |  |  |  |

**APN mortality table -B**

| Source | DF | SS | MS | F | P |
| --- | --- | --- | --- | --- | --- |
| V002 | 1 | 5589.2 | 5589.24 | 12.73 | 0.0016* |
| V001 | 2 | 4141.9 | 2070.95 | 4.72 | 0.0187* |
| V002* V001 | 2 | 874.2 | 437.12 | 1.00 | 0.3844 |
| Error | 24 | 10540.3 | 439.18 |  |  |
| Total | 29 | 21145.7 |  |  |  |

Voo2: Time intervals (6, 12, 15 DAT)

V001: Control ( ds RNA dilution Buffer) and Treatment (ds RNA of Cyp)

V002 * V001 : Interaction between treatment and time course

DF; degrees of freedom

SS: Sum of squares

MS: mean squares

F: F value

P: probability , * significant
