# Supplementary material for: Silencing of CYP6 and APN Genes Affects the Growth and Development of Rice Yellow Stem Borer, Scirpophaga incertulas
Source: Front Physiol. 2016 Feb 12;7:20. doi: 10.3389/fphys.2016.00020 (PMC4751738; doi:10.3389/fphys.2016.00020)
Supplement: Supplementary file 2 [file Table2.doc]

**Supplementary table - 2**

**(A)Effect of dsRNA *APN* on PSB larvae**

**1. Larval length**

| **Time intervals** | **Control**  (Mean ±SE) | **Treatment**  (Mean ±SE) | **T value** | **P value** | **Significant/ NS**  **5 %L** |
| --- | --- | --- | --- | --- | --- |
| 6 DAT | 0.64 ± 0.08 | 0.47 ± 0.08 | 1.51 | 0.1664 | NS |
| 12 DAT | 1.35 ± 0.15 | 1.57 ± 0.2 | -0.71 | 0.5269 | NS |
| 15 DAT | 1.65 ± 0.06 | 1.70 ± 0.1 | -0.45 | 0.6985 | NS |

**2. Larval weight**

| **Time intervals** | **Control**  (Mean ±SE) | **Treatment**  (Mean ±SE) | **T value** | **P value** | **Significant/ NS**  **1 % L** |
| --- | --- | --- | --- | --- | --- |
| 6 DAT | 2.6 ± 0.8 | 3.0 ± 1.4 | -0.23 | 0.8220 | NS |
| 12 DAT | 29.5 ± 0.5 | 29.0 ± 1.0 | 0.37 | 0.7345 | NS |
| 15 DAT | 49.0 ± 1.0 | 30.0 ± 1.0 | 13.44 | 0.0055 | ***** |

**(B)Effect of dsRNA *CYP6* on PSB larvae**

**1. Larval length**

| **Time intervals** | **Control**  (Mean ± SE) | **Treatment**  (Mean ± SE) | **T value** | **P value** | **Significant/ NS**  **5 % L** |
| --- | --- | --- | --- | --- | --- |
| 6 DAT | 0.64 ± 0.08 | 0.90 ± 0.1 | -1.78 | 0.1348 | NS |
| 12 DAT | 1.35 ± 0.15 | 1.47 ± 0.2 | -0.97 | 0.4020 | NS |
| 15 DAT | 1.65 ± 0.05 | 1.73 ± 0.1 | -0.83 | 0.4929 | NS |

**2. Larval weight**

| **Time intervals** | **Control**  (Mean ±SE) | **Treatment**  (Mean ± SE) | **T value** | **P value** | **Significant/ NS**  **at 5 % L** |
| --- | --- | --- | --- | --- | --- |
| 6 DAT | 2.6 ± 0.8 | 3.0 ± 1.0 | -0.27 | 0.7949 | NS |
| 12 DAT | 29.5 ± 0.5 | 29.0 ± 1.0 | 0.45 | 0.6985 | NS |
| 15 DAT | 49.0 ± 1.0 | 48.75 ± 0.3 | 0.24 | 0.8310 | NS |

NS: Non significant; * indicates- significance
