# Supplementary figures and images for: Silencing of CYP6 and APN Genes Affects the Growth and Development of Rice Yellow Stem Borer, Scirpophaga incertulas
Source: Front Physiol. 2016 Feb 12;7:20. doi: 10.3389/fphys.2016.00020 (PMC4751738; doi:10.3389/fphys.2016.00020)

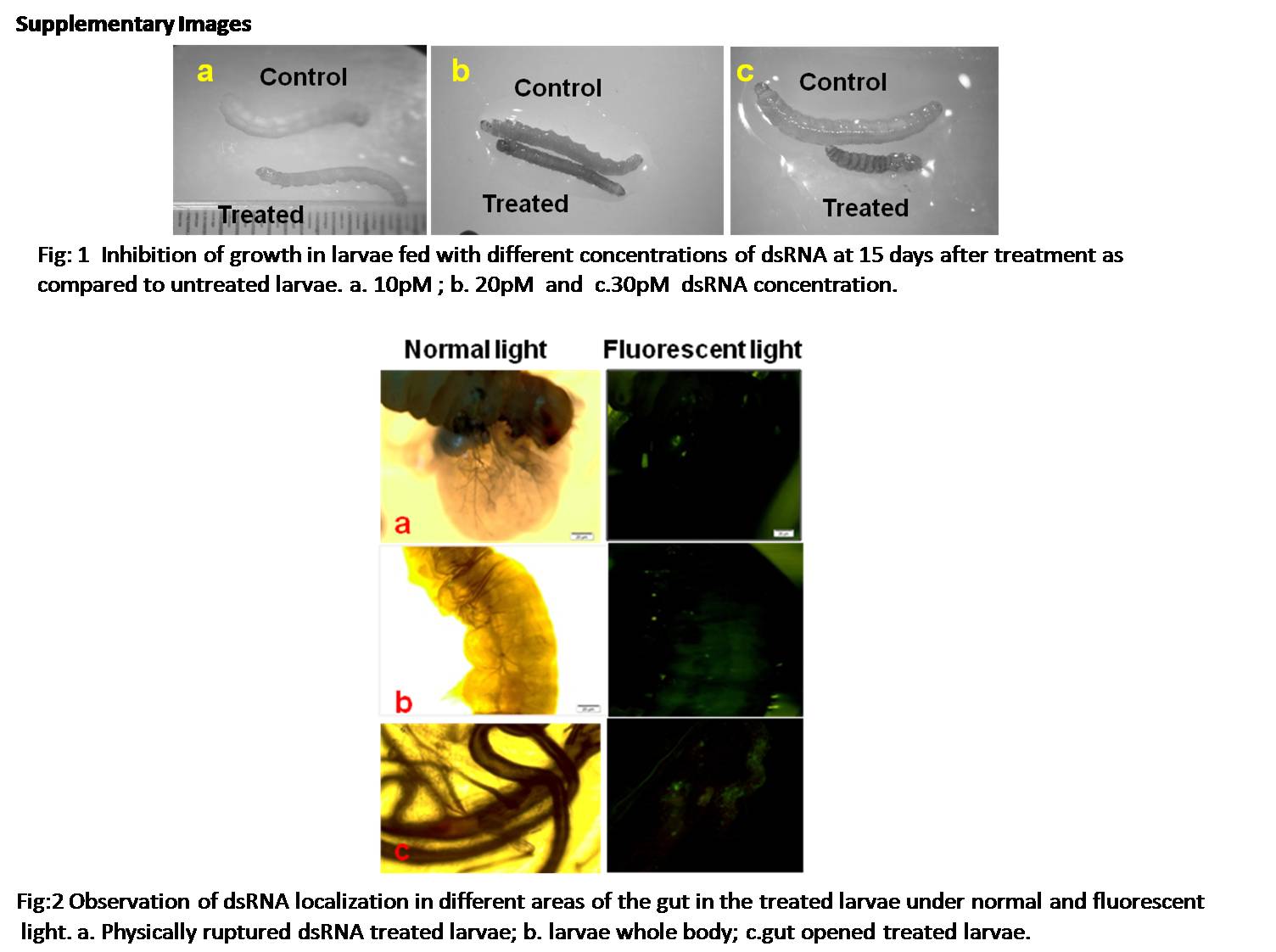

Supplement: Supplementary file 6 [file Image1.JPEG]
